# Nefopam Misuse: A Cross‐Sectional Study in France

**DOI:** 10.1002/ejp.70083

**Published:** 2025-07-20

**Authors:** E. Barat, E. Lacroix, A. Gillibert, B. Gerard, P. Brevet, R. Leguillon, S. Pouplin, J. Grosjean, R. Varin

**Affiliations:** ^1^ Department of Pharmacy CHU de Rouen Rouen France; ^2^ Normandie University, UNICAEN, Inserm U1086 Caen France; ^3^ Department of Biostatistics CHU Rouen, CEDEX Rouen France; ^4^ Department of Rheumatology CHU Rouen, CEDEX Rouen France; ^5^ Laboratoire D'informatique Médicale et D'ingénierie Des Connaissances en e‐Santé (LIMICS), U1142, INSERM Sorbonne Université Paris France; ^6^ Department of Digital Health CHU Rouen Rouen France; ^7^ Sorbonne Université Paris France; ^8^ Department of Pharmacy UNIROUEN, Inserm U1234 Rouen France; ^9^ Normandie University Rouen France

## Abstract

**Background:**

Nefopam is a non‐opioid analgesic. Nefopam is increasingly prescribed in contraindicated populations, raising questions about the appropriate use of nefopam. This study aimed to evaluate nefopam use in France according to age, epilepsy status and socioeconomic status.

**Methods:**

This retrospective observational study was conducted from January 1, 2016, to December 31, 2022. Data on nefopam use in France were retrieved from the national health insurance information system.

**Results:**

Between 2016 and 2022, 51.8 million boxes of nefopam were dispensed to 6.5 million patients, with a mean age of 56 years and a male‐to‐female sex ratio of 0.6. Nefopam use increased by 121.7%, whereas codeine use and tramadol use decreased by 7% and 17% respectively, raising the market share of nefopam from 5.0% to 11.6%. Nefopam use increased 2.8‐fold in patients under 15 years and 1.7‐fold in patients over 65 years, after standardisation. Nefopam use increased 1.7‐fold in patients with epilepsy and 1.2‐fold in patients with a disadvantaged socioeconomic status. The cost of nefopam was 4.3 times higher than alternative drugs. During the period 2016–2022, 8.23% of the general population received 1 to 4 boxes of nefopam per year, 4.68% received 6 to 19 boxes per year, and 0.91% received ≥ 20 boxes per year.

**Conclusions:**

This study highlights a widespread inappropriate use of nefopam, especially in contraindicated populations and vulnerable groups. The recent availability of the oral form in France may worsen these patterns. Close national and European monitoring is important to assess the extent of such practices and guide future regulation.

**Significance Statement:**

This work is a snapshot of nefopam usage in France. It sheds light on contraindicated uses (in subjects under 15 years of age or with epilepsy), not recommended uses (chronic usage, or in the elderly) and reports the usage of this treatment in the most deprived patients. This article cautions against underestimating the dangers and pitfalls of this treatment, especially in light of mounting evidence of pharmacodependence.

## 1 Introduction

Nefopam is a non‐opioid analgesic indicated for the management of acute pain, in particular post‐surgical pain. In France, until recently, nefopam was only available as an intravenous formulation. The absence, in France, of an oral formulation led to the off‐label use of the intravenous formulation, raising questions about prescribing practices. (Schulz et al. 2022).

In addition to the oral use of the intravenous formulation, nefopam is widely used for the long‐term management of pain. The advantage of nefopam is that it is not an opioid analgesic, and thus patients are not exposed to the various risks associated with opioid misuse. Today, nefopam is subject to strict prescription rules. It cannot be prescribed to patients under the age of 15 years, to patients with epilepsy, and is reserved for the management of acute pain. However, it has anticholinergic properties responsible for numerous adverse effects, particularly in the elderly, such as confusion, urinary retention, constipation and hyposalivation leading to malnutrition. Nefopam is also contraindicated in patients with closed‐angle glaucoma or prostate hypertrophy, which are common conditions in the elderly (Bannwarth 2004). Therefore, nefopam is not recommended in elderly patients. Finally, there is a risk of nefopam misuse (Revol et al. 2021). In general, the most disadvantaged populations are those most exposed to misuse, and this is particularly true for opioid misuse (Brady et al. 2023; Cesare et al. 2024), (Garcia‐Larrea and Bastuji 2018). Therefore, it seems relevant to evaluate nefopam use among disadvantaged populations.

In France, all healthcare data, including drug prescriptions, are stored in a national health data system (SNDS: Système National des Données de Santé).

To date, no study has evaluated nefopam prescribing in France or the conformity of nefopam prescriptions to various guidelines. The main objective of this study was to evaluate nefopam prescribing and the conformity of nefopam prescriptions to different guidelines. The secondary objective was to evaluate nefopam prescribing according to age, epilepsy status and socioeconomic status.

## 2 Methods

This retrospective observational study was conducted from January 1, 2016, to December 31, 2022. Data on nefopam use in France were retrieved from the national health insurance information system (SNIIRAM: Système National d'Information Inter‐Régimes de l'Assurance Maladie), a component of the SNDS.

The SNIIRAM is a repository of anonymous data that includes information on reimbursements made by all health insurance schemes in France (1.2 billion claims for the entire population living in France).

Data on the beneficiary include age, sex, and place of residence (department and municipality), as well as, where applicable, the notion of eligibility for universal health coverage, long‐term conditions (LTC), or occupational diseases, and, if applicable, the date of death. Data on healthcare professionals who provide care and possibly prescriptions include age, sex and place of practice, as well as specialty and mode of practice. Data on healthcare use include the dates of treatment as well as the cost incurred by the patient and the cost reimbursed by the health insurance scheme. It also includes detailed coding of dispensed medications, medical procedures, medical devices and laboratory sampling.

In this study, age, epilepsy status, socioeconomic status and the long‐term use of nefopam were evaluated. A disadvantaged socioeconomic status was defined as eligibility for « CMU » (Universal Health Coverage) or « AME » (State Medical Assistance). The long‐term use of nefopam was defined as at least 3 boxes of nefopam during 6 months. Among patients with a long‐term use of nefopam, we searched the proportion of patients with a LTC. Additionally, we compared the number of patients prescribed nefopam in France and the total eligible population in France, that is, the population aged over 15 years.

To determine which patients had a disadvantaged socioeconomic status, we first searched the “pharmacy” table of the SNDS to identify the number of nefopam prescriptions by year using the ATC code N02BG06. Then, we crosslinked with the comprehensive table of benefits of the SNDS to identify the number of beneficiaries of nefopam by year (by counting distinct beneficiary identifiers in the SNDS) and the number of boxes of nefopam dispensed by year.

The difference in cost between an ampoule of nefopam (the only formulation available in France between 2016 and 2022) and the mean cost, 95% confidence interval, of the following drugs: lamaline, izalgi, paracetamol 1 g, tramadol 50 mg, and paracetamol/codeine 500 mg/30 mg—was retrieved. The difference in the number of codeine, codeine/paracetamol, tramadol, tramadol/paracetamol, opium‐containing and nefopam prescriptions between 2016 and 2022 was retrieved.

This study was observational and evaluated patients who were prescribed nefopam at least once. No comparative statistical analysis was performed as part of this study; however, age and sex were standardised based on the INSEE (Institut National de la Statistique et des Etudes Economiques) population in 2022 in France.

After aggregation of nefopam dispensing by year (from 2016 to 2022), linear trends of nefopam dispensing were assessed by simple linear regressions explaining nefopam dispensing by year.

## 3 Results

From January 1st, 2016, to December 31st, 2022, 51.8 million boxes of nefopam were dispensed to 6.5 million patients. The mean age of patients was 56 years, with a mean sex ratio (M/F) of 0.6. The number of patients prescribed nefopam increased 2.1‐fold compared with the standardised rate based on the INSEE population in 2022, as shown in Table 1.

**TABLE 1:** Nefopam consumption trends over the years; * nefopam general population standardised rate by age group and sex, based on INSEE population structure in 2022.

year	2016	2017	2018	2019	2020	2021	2022	slope/year (95% CI)
Number of boxes dispensed (thousands)	5168	5989	6751	7477	8044	8769	9606	720 (683 to 758)
Number of patients (thousands)	591	700	809	937	1000	1162	1311	117 (104 to 130)
Average number of boxes per patient per year	8.7	8.6	8.3	8	8	7.5	7.3	−0.24 (−0.29 to −0.19)
Sex ratio	0.6	0.6	0.6	0.6	0.6	0.6	0.6	
Mean age	59	57	57	56	56	55	54	−0.71 (−0.95 to −0.48)
Standardised * rate of nefopam use	1.80%	2.09%	2.43%	2.80%	2.97%	3.44%	3.85%	0.34% (0.30% to 0.37%)
Standardised * rate of nefopam use with epilepsy	2.34%	2.77%	2.98%	3.33%	3.45%	3.68%	4.04%	0.26% (0.22% to 0.30%)
Standardised * rate of nefopam use with < 15 years old (/100,000)	2	3	3	6	6	10	9	1.4 (0.8 to 1.9)

In comparison with other drugs, the number of nefopam prescriptions increased by 121.7%, whereas the number of codeine (with or without paracetamol) and tramadol (with or without paracetamol) prescriptions decreased by 7% and 17%, respectively. The share of nefopam relative to tramadol and codeine increased from 5.0% to 11.6% (Table 2).

**TABLE 2:** Changes in the consumption of codeine, codeine/paracetamol, tramadol, tramadol/paracetamol, opium and nefopam between 2016 and 2022.

Drug	2016	2017	2018	2019	2020	2021	2022	slope/year (95% CI)
Codeine (thousands)	28	27	24	24	23	24	23	−0.90 (−1.39 to −0.40)
Codeine in combination (thousands)	4826	4826	4659	4500	4346	4420	4489	−76 (−129 to −23)
Tramadol in combination (thousands)	3276	3043	2805	2515	2217	2115	1980	−226 (−262 to −190)
Tramadol (thousands)	3282	3448	3548	3531	3268	3430	3487	10.8 (−47.9 to 69.4)
Opium (thousands)	297	296	310	317	331	334	366	10.9 (6.9 to 14.9)
Nefopam (thousands)	591	700	809	937	1000	1162	1311	117 (104 to 130)
Total (thousands)	12,301	12,340	12,156	11,824	11,186	11,485	11,657	−165 (−300 to −29)

**FIGURE 1:**
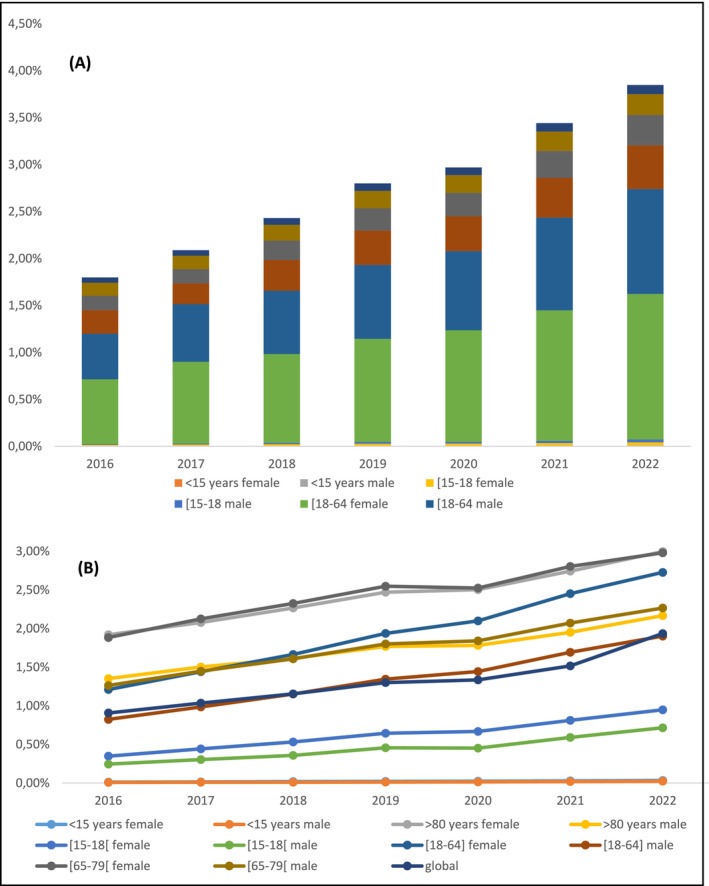
(A) Cumulative standardised rate of nefopam consumption in the general population, adjusted to the 2022 INSEE age and sex distribution. (B) Age‐ and sex‐stratified standardised rates of nefopam consumption across population subgroups.

Among patients under the age of 15 years, a 2.72‐fold increase was observed in the absolute number of patients prescribed nefopam, and a 2.8‐fold increase after standardisation. Similarly, among patients over the age of 64 years, a 2.07‐fold increase was observed in the absolute number of patients prescribed nefopam, and a 1.7‐fold increase after standardisation (Figure 1A). Considering subgroup populations, the highest proportions of patients prescribed nefopam were women and men over the age of 64 years, and women aged 18 to 64 years (Figure 1B). Among patients prescribed nefopam, a mean of 8.5% had a disadvantaged socioeconomic status, compared to 8.4% in the general population.

**FIGURE 2:**
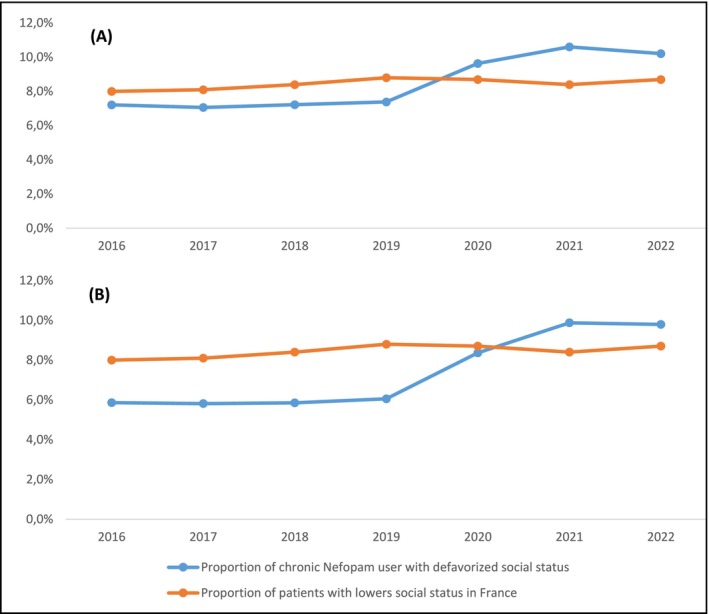
(A) Proportion of patients with disadvantaged socioeconomic status among nefopam users, compared to the proportion in the general population in France. (B) Proportion of patients with disadvantaged socioeconomic status among chronic nefopam users, compared to the proportion in the general population in France.

Since 2020, a higher proportion of patients with a disadvantaged socioeconomic status were prescribed nefopam compared to the general population (Figure 2A). Since 2021, a higher proportion of patients with a disadvantaged socioeconomic status had a long‐term prescription of nefopam compared to the general population (Figure 2B).

**FIGURE 3:**
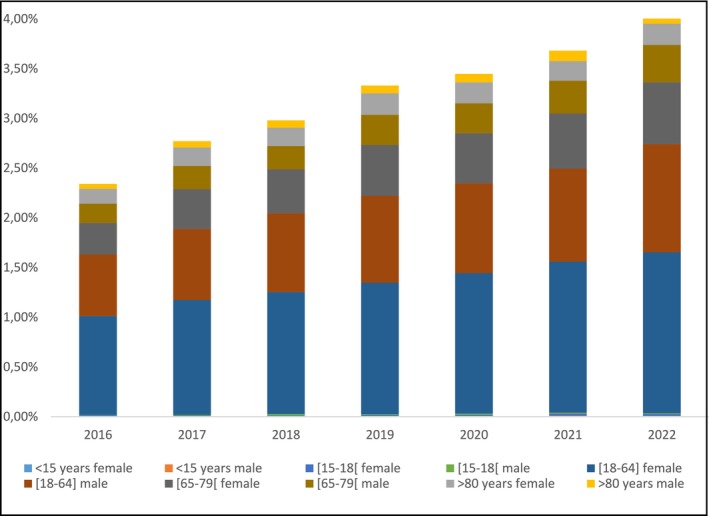
Standardised rate of nefopam prescriptions among individuals with epilepsy, by year, age, and sex, adjusted to the 2022 INSEE age and sex distribution.

The mean cost per tablet of lamaline, izalgi, paracetamol, tramadol and paracetamol/codeine was €0.12 [CI €0.09; €0.14], compared to €0.52 for an ampoule of nefopam, 4.3 times higher than alternative drugs. Among patients with a diagnosis of epilepsy, a 1.7‐fold increase was observed in the number of patients prescribed nefopam after standardisation, compared to a 1.4‐fold increase in the general population. In 2022, the proportion of nefopam prescriptions in patients with epilepsy was similar to that in the general population (Figure 3).

**FIGURE 4:**
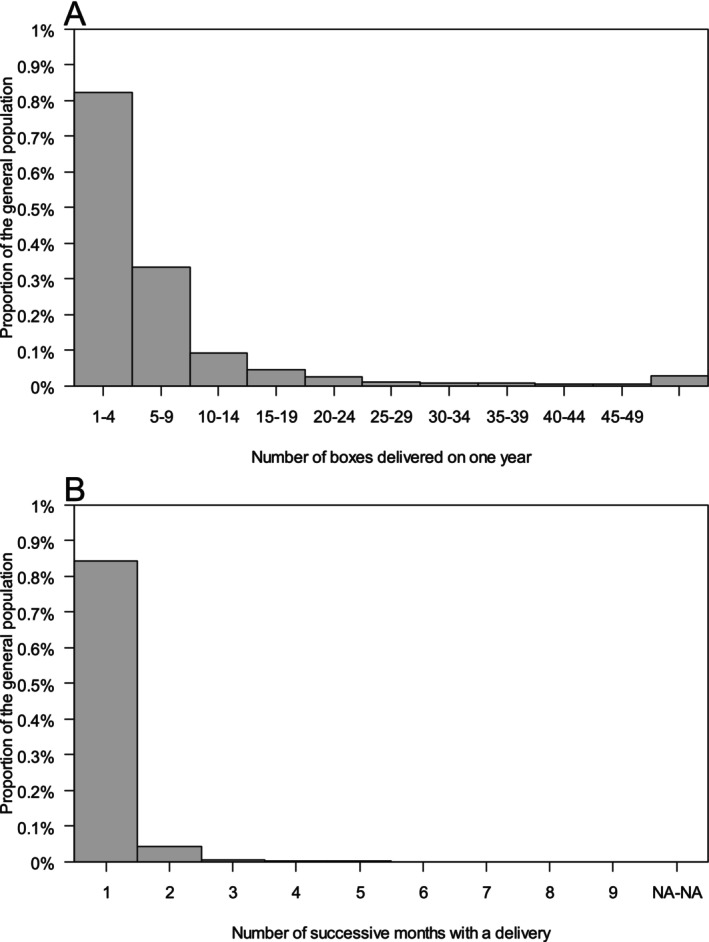
Histogram of consumption of nefopam in the overall French population. Panel A shows the proportion of the general population and the number of boxes of nefopam they received per year (from January to December) computed as the average of 7 years (2016 to 2022) and panel B shows the proportion of the general population and the number of months they received nefopam during the period 2016–2022.

During the period 2016–2022, 8.23% of the population received 1 to 4 boxes of nefopam per year, 4.68% received 6 to 19 boxes per year, and 0.91% received ≥ 20 boxes per year (Figure 4A). (Figure 4B). During the period, 8.4% of the general population received nefopam for 1 month, 0.4% for 2 months, 0.08% for 3 to 5 months, and 0.013% for 6 months or more.

## 4 Discussion

This study showed a significant increase in nefopam use between 2016 and 2022. Besides the fact that the number of patients prescribed nefopam more than doubled over 7 years, we also observed a positive incidence every year, indicating a consistent year‐on‐year increase. Several hypotheses can be proposed to explain this increase. First, the withdrawal of dextropropoxyphene in 2011 may have triggered the rise in nefopam use, while its classification as a non‐opioid analgesic and its compatibility with paracetamol may have sustained this trend. Second, shifts in healthcare models in France, such as the development of outpatient surgery and the administration of nefopam at home by private nurses, may account for the significant growth in nefopam use. Conversely, although the COVID‐19 pandemic profoundly disrupted access to healthcare and prescribing practices, it appears to have had a minimal impact on nefopam use.

In accordance with the marketing authorisation (AMM) for nefopam, prescription should be restricted to acute pain. In a recent study, authors described a risk of nefopam misuse (Revol et al. 2021). Although we identified a lower long‐term use of nefopam than Révol et al., our results remain comparable in terms of proportion. This risk of misuse could be partly explained from a physiological standpoint. Indeed, the areas of the tertiary cortex of the ‘pain matrix’ are highly plastic and sensitive to numerous factors, including pharmacological substances, such as nefopam, a centrally acting analgesic (Garcia‐Larrea and Bastuji 2018; Garcia‐Larrea and Peyron 2013). A significant long‐term use of nefopam outside recommendations could partly explain the increase in nefopam addiction. This increase in long‐term nefopam use is all the more harmful as it mainly involves older patients. In the subgroup analysis, we found that the oldest age groups exhibited the highest rates of long‐term nefopam use. Specifically, among nefopam users aged over 80 years in 2022, more than one in four women and nearly one in five men were long‐term users.

In older people, nefopam was clearly identified as an anticholinergic drug, making it a potentially inappropriate medication (PIM) to avoid in the elderly, in accordance with the STOP/START V.3 list (O'Mahony et al. 2023). It is noteworthy that nearly 30% of nefopam prescriptions involved patients aged over 65 years. In sub‐group populations, patients over 65 years were the most impacted by the long‐term prescribing of nefopam. There is therefore a significant use of nefopam in the elderly outside recommendations. This observation is even more concerning considering that the oral route is preferred (Schulz et al. 2022), and oral use leads to significant hepatic first‐pass effects, which lead to an increased frequency of adverse effects (Aymard et al. 2003). This could have a negative impact on the quality of life of older people (Fox et al. 2011; Kalisch Ellett et al. 2014).

Nefopam is contraindicated in patients under the age of 15 years. However, it continues to be prescribed in this population, mostly occasionally but also long term. It is likely that the oral use of the intravenous formulation has facilitated the excessive use of nefopam despite its contraindication. Similarly, there is a constant increase in nefopam use in individuals with epilepsy, and in 2022, the proportion of nefopam use in patients with epilepsy was similar to that in the general population.

The increase in nefopam use appears to correspond to a decrease in the use of weak opioids. At first glance, this use of nefopam seems beneficial in a context of opioid sparing. However, opioid sparing should not come at the cost of using non‐recommended or even contraindicated drugs such as nefopam. Furthermore, in certain populations, particularly the elderly, nefopam is not recommended (Capriz et al. 2017); (Rodger et al. 2015). Conversely, tramadol in drop form is considered ‘the most manageable opioid’ in the elderly (American Geriatrics Society Panel on Pharmacological Management of Persistent Pain in Older Persons 2009). Thus, nearly 30% of nefopam prescriptions may not be justified. It is also worth noting that the efficacy of nefopam, especially via oral administration, is not universally supported in the literature (Moore et al. 2015). Nefopam most likely has a role in the therapeutic arsenal for pain management, including in efforts to combat opioid misuse. However, its use must be rational and carefully monitored.

This study highlights a significant impact of increased nefopam prescribing in recent years in the most socially vulnerable patients. Indeed, we observed a constant increase in the proportion of patients with CMU and a nefopam prescription in absolute value, whether in occasional or long‐term use. Furthermore, we observed a higher proportion of patients with CMU among nefopam users than in the French population. There are two explanations: one positive, namely that pain in socially vulnerable patients is better managed, and the second hypothesis is more negative, namely that the increase in nefopam prescription exposes socially vulnerable patients to inappropriate prescriptions more rapidly. The second explanation is more likely given the various elements raised in this study (use in elderly people, in young people under 15 years, and long‐term use), and the literature on social health inequalities (Reyes et al. 2023). However, it is interesting to note that the cost factor is not considered. In fact, nefopam is 4.3 times more expensive than other analgesic treatments.

This study, while informative, has certain limitations. Firstly, the SNDS database reflects nefopam dispensations and not nefopam use. Secondly, we assume that long‐term use corresponds to excessive use outside the indications of marketing authorisation for nefopam.

In several countries such as the United Kingdom, Belgium, Luxembourg, India, and China, oral formulations of nefopam have been available for many years, and global market projections anticipate a continued rise in its use (PMarketResearch 2025). The recent introduction of oral nefopam in France may lead to an even sharper increase in national consumption, potentially intensifying the inappropriate use patterns identified in our study, which was conducted just prior to its release. It therefore seems important to closely monitor nefopam use in the coming years to detect and address any emerging deviations.

This study highlights a potential public health concern, with an increasing number of nefopam prescriptions and a significant proportion of prescriptions outside any guidelines. The main explanation likely lies in the oral use of intravenous formulations. In addition to highlighting a public health concern, this study underlines the need for further research on nefopam, particularly regarding the clinical efficacy of the different formulations, whether intravenous or oral, as well as the long‐term use of nefopam in pain management. However, nefopam use in patients under 15 and over 80 years should be more closely monitored, especially by community pharmacists.

## 5 Conclusion

This study demonstrates a high prevalence of nefopam use in contraindicated populations (notably those under 15 years and individuals with epilepsy), substitution patterns following opioid regulation, and unequal exposure among socially vulnerable groups. These findings raise significant public health concerns, particularly with the recent introduction of the oral formulation of nefopam in France, which may facilitate access and exacerbate inappropriate use. Close national monitoring is warranted. Furthermore, a coordinated European surveillance effort appears relevant, given the likely rise in global consumption. Such monitoring would help determine whether similar issues exist elsewhere in Europe or whether controlled access to oral nefopam mitigates these risks. Comparative insights are crucial for guiding future regulation and clinical practice.

## Author Contributions

This study was designed by E.B., B.G., and E.L. The experiments were performed by E.B., R.L. and E.L. The data were analysed by E.B., A.G., S.P. and E.L., and the results were critically examined by all authors. The study was supervised by R.V. and A.G. E.B. had a primary role in preparing the manuscript. All authors have approved the final version of the manuscript and agree to be accountable for all aspects of the work.
